# Treatment gap and barriers for mental health care: A cross-sectional community survey in Nepal

**DOI:** 10.1371/journal.pone.0183223

**Published:** 2017-08-17

**Authors:** Nagendra P. Luitel, Mark J. D. Jordans, Brandon A. Kohrt, Sujit D. Rathod, Ivan H. Komproe

**Affiliations:** 1 Research Department, Transcultural Psychosocial Organization (TPO) Nepal, Kathmandu Nepal; 2 Centre for Global Mental Health, Institute of Psychiatry, Psychology and Neuroscience, King’s College London, London, United Kingdom; 3 Research and Development Department, HealthNet TPO Amsterdam, Amsterdam, the Netherlands; 4 Duke Global Health Institute, Department of Psychiatry and Behavioral Sciences, Duke University School of Medicine, Durham, United States of America; 5 Centre for Global Mental Health, Department of Population Health, London School of Hygiene and Tropical Medicine, London, United Kingdom; 6 Faculty of Social and Behavioural Sciences, Utrecht University, Utrecht, the Netherlands; Yokohama City University, JAPAN

## Abstract

**Context:**

There is limited research on the gap between the burden of mental disorders and treatment use in low- and middle-income countries.

**Objectives:**

The aim of this study was to assess the treatment gap among adults with depressive disorder (DD) and alcohol use disorder (AUD) and to examine possible barriers to initiation and continuation of mental health treatment in Nepal.

**Methods:**

A three-stage sampling technique was used in the study to select 1,983 adults from 10 Village Development Committees (VDCs) of Chitwan district. Presence of DD and AUD were identified with validated versions of the Patient Health Questionnaire (PHQ-9) and Alcohol Use Disorder Identification Test (AUDIT). Barriers to care were assessed with the Barriers to Access to Care Evaluation (BACE).

**Results:**

In this sample, 11.2% (N = 228) and 5.0% (N = 96) screened positive for DD and AUD respectively. Among those scoring above clinical cut-off thresholds, few had received treatment from any providers; 8.1% for DD and 5.1% for AUD in the past 12 months, and only 1.8% (DD) and 1.3% (AUD) sought treatment from primary health care facilities. The major reported barriers to treatment were lacking financial means to afford care, fear of being perceived as “weak” for having mental health problems, fear of being perceived as “crazy” and being too unwell to ask for help. Barriers to care did not differ based on demographic characteristics such as age, sex, marital status, education, or caste/ethnicity.

**Conclusions:**

With more than 90% of the respondents with DD or AUD not participating in treatment, it is crucial to identify avenues to promote help seeking and uptake of treatment. Given that demographic characteristics did not influence barriers to care, it may be possible to pursue general population-wide approaches to promoting service use.

## Background

Globally, there is a significant gap between the number of individuals in need of mental health care and those who actually receive treatment, with prior estimates suggesting that more than 56% of persons with depression [1–4] and 78% of persons with alcohol abuse and dependence [3] have not received care. A study of 21 countries with the World Health Organization (WHO) Mental Health Surveys found that 52.6% of persons with depressive disorder in low-income countries received any treatment in the past 12 months, and only 20.5% of persons with depressive disorder received minimally adequate treatment [5]. Studies have documented several adverse consequences of untreated mental illness including pre-mature mortality [6, 7], unemployment [8, 9], poverty [10], homelessness [11], co-morbid substance abuse and addiction [12, 13], poor physical health [14–16] and suicide [17]. Some initiatives have been taken recently to reduce the treatment gap for mental health care [18–22]. However, the gap is still high, especially in low and middle-income countries (LMICs) [1, 3, 4, 23]. Understanding the reasons people with mental disorders drop out of or fail to seek treatment could help in developing policies and plans to reduce these barriers to mental health treatment. Various factors are considered to impede mental health treatment including lack of perceived need, stigma, not knowing where to go for treatment, belief that the problem will resolve itself, desire to deal with the problem oneself, inability to afford treatment expenses, doubt regarding the effectiveness of the treatment, and lack of services [24–29].

In Nepal, few studies have been conducted on mental health. Most prior studies have focused on the mental health problems of populations affected by armed conflict, and none have attempted to estimate the treatment gap for mental health care or identify potential barriers to treatment [30–32]. In addition, there is a scarcity of population-wide mental health services in Nepal. Existing mental health resources are allocated unequally; mental health services are restricted to a small number of hospitals located in few big cities [33]. This study was conducted as a part of PRIME (PRogramme for Improving Mental health carE) research program consortium, which aims to study the implementation and scaling up of treatment programs for priority mental disorders in the primary health care context [34]. In this study we aimed to assess the treatment gap, as we expected this to be even more pronounced than in other LMICs [4], and to better understand the barriers that contribute to the treatment gap, anticipating that these barriers are more prominent in a fragile-state setting [35, 36]. The aims of this paper are to describe the treatment gap among adults with DD and AUD, examine possible barriers to initiating mental health treatment, and investigate demographic predictors of reporting treatment barriers in Nepal.

## Materials and methods

This study was conducted as a part of PRIME research program consortium [34] to estimate the prevalence and treatment contact coverage for DD and AUD in four LMICs (Nepal, India, Ethiopia and Uganda) [37]. Details of the PRIME evaluation methodology can be found in a separate publication [38].

### Setting

This study was conducted in Chitwan, a district in southern Nepal. Nepal, one of the poorest countries in South Asia, has a total population of approximately 26.4 million with 69.1 years life expectancy at birth. The United Nations ranks Nepal 145^th^ out of the world’s 188 countries on the Human Development Index (HDI) [39]. The total population of Chitwan is 579,984 (279,087 male and 300,897 female) with about 132,462 households. The literacy rate of Chitwan district is 78.9%, which is higher than the national average of 67% [40]. In Nepal, mental health services are restricted to a few government hospitals located in big cities and private hospitals; however, in Chitwan mental health services including treatment for AUD (both inpatient and outpatient services) are also available in the district hospital and medical colleges operating in the district. Evidence suggests that the availability of specialized mental health services alone will not be effective in minimizing the treatment gap [2]. Involvement of primary health care (PHC) workers in detection and management of mental health problems has been recommended as one of the most effective and resource-efficient strategies for reducing the treatment gap for mental heath care, especially in the LMICs [21]. Therefore, we selected Chitwan district in order to assess the treatment gap and barriers to initiating treatment among a community sample where specialists services are available in the district hospital and private hospitals.

### Study participants and sampling

A three-stage sampling technique was used in the study to select one adult from each of 1983 households across 90 wards in Chitwan district. Information about the ward populations was collected from the Village Development Committees, which are the local governing structures below the district level. First, the total target sample was stratified in proportion to the population size of each of the 90 wards. Second, households from each ward were selected using a systematic random sampling technique. The field workers then used a (simple) random selection procedure to select one adult from each household. In this procedure, the field workers first prepared a list (roster) of all members living in each household including names, age, sex and so forth. The survey inclusion criteria of age 18 years or above, resident of the implementation area, ability to provide informed consent and fluency in the Nepali language was applied and a separate list was prepared from the roster to reflect the inclusion criteria. Finally, a member of each household drew a name of an eligible participant from within that household. If no one was found at the household after three visits, or no one was willing to participate in the study or the selected adult was not willing to participate in the study, then the interviewers would go to the nearest neighboing household to assess its members for the inclusion criteria.

### Instruments

The questionnaire administered to participants had several sections, including: sociodemographic characteristics, screening for depression, screening for alcohol use disorder, suicide ideation and behaviors, mental health treatment history, barriers for mental health treatment, disability, and mental health literacy. Details of all the instruments used in the study have been presented elsewhere [37]. The instruments used in gathering data reported in this paper are described below.

#### Patient health questionnaire (PHQ9)

The PHQ9 is a self-reported screening tool designated for use in various medical settings. PHQ9 has been widely used and validated in primary care, medical outpatient, and specialist medical services [41]. It has nine items and the respondents are asked to score their experience of nine common symptoms of depression in the past 2 weeks. It has a 4-point rating scale where 0 indicates ‘not at all’ and 3 indicates ‘always’. The PHQ9 has been translated and validated in Nepal. A cutoff score of ≥10 has sensitity of 0.94 and specificity of 0.80 to detect a current episode of moderate to severe depression [42]. After completing the PHQ9, each participant was asked an additional question to assess depressive episodes in the past 12 months period. Participants were asked, “Apart from these past two weeks, during the past 12 months, did you have other episodes of two weeks or more when you felt depressed or uninterested in most things, and had most of the problems we just discussed about?” We considered those with an affirmative response to the additional question or a score of 10 or more on the PHQ9 to have depressive disorder.

#### Alcohol use disorder identification test (AUDIT)

AUDIT was developed by World Health Organization (WHO) as a screening tool in primary health care [43]. AUDIT has been validated in Nepal using DSM-IV diagnostic categories (alcohol use and alcohol dependence) as the gold standard to calculate the diagnostic parameters of the AUDIT and a cutoff score of ≥9 has been recommended for alcohol dependence or alcohol abuse for both males (sensitivity 96.7, specificity 91.7) and females (sensitivity 94.3, specificity 91.4) [44].

#### Barriers to Access to Care Evaluation (BACE)

BACE scale was originally developed by involving both experts and service users [45]. The BACE is a 30-item self-reported instrument, where respondents are asked whether each listed barrier has ever stopped, delayed or discouraged them from receiving or continuing care for their mental health problems. It includes some barriers related to stigma and discrimination. BACE has been translated and validated in other settings [46]. It has a four-point response scale ranging from 0 (‘not at all’) to 3 (‘a lot’). Results for each barrier can be presented in three ways: mean score for the item, barrier to any degree (i.e. the percentage circling 1, 2 or 3) or major barrier (i.e. the percentage circling 3). We followed a systematic approach that has been developed in Nepal for translation and adaptation of standardized tools for translation and contextualization of BACE in Nepali language [47]. In addition, we conducted pilot testing of the Nepali version of BACE with different populations (such as general community members and the help seeking population in primary health care facilities) to assess its understandability and acceptability in the Nepalese context. Furthermore, we found excellent internal consistency (i.e. Cronbach’s alpha 0.91) of BACE in the sample.

#### Treatment contact coverage for depression or AUD

Respondents who were considered to have depression or alcohol use disorder were subsequently asked whether they had sought treatment for that disorder in the past 12 months. A cut off score of ≥10 in PHQ9 and a cut off score of ≥ 8 in AUDIT were set in the device application used for data collection; hence, the devices automatically directed field workers to continue the subsequent treatment section with those who scored above these cut off points. Service providers were categorized into three groups: specialist mental health providers (psychiatrist, psychologist, counsellor, mental health nurse), generalist health providers (medical doctor, health assistant, auxiliary health worker, community medical assistant), or traditional providers (traditional healer or religious leader). In this study, contact coverage is defined using the framework described by Tanahashi [48], which is the proportion of individuals with depressive disorder or alcohol use disorder who accessed a health care provider for that condition in the past one year.

### Procedure

The interviewers were twelve Nepali-speaking research assistants who had completed an undergraduate degree. Two months of training covering the topics of interviewing skills, rapport building, informed consent, inclusion/exclusion criteria and content of the questionnaire, as well as field testing were organized. Android tablets with a questionnaire application were used for data collection. The interviews were conducted in the respondents’ place of residence or in a confidential place upon respondents’ request. The study received ethical approval from the ethical review board of the World Health Organization (WHO) and the Nepal Health Research Council (NHRC). Written informed consent was acquired from literate study participants to enrol in the study. As taking fingerprints is not always culturally appropriate in Nepal, only verbal informed consent was obtained from the illiterate participants. Selections of the dataset may be made available to researchers via the PRIME consortium Expression of Interest form, which is available at http://www.prime.uct.ac.za/contact-us.

### Analysis

First, the design-based analysis was adjusted for the stratified sampling procedure, where households were randomly selected from within strata of VDCs (Village Development Committee) and wards. Participant data was weighted according to the inverse probability of sampling (i.e. 1 / (probability of selecting a household within the ward X probability of selecting an adult within the household). We report frequency of socio-demographic characteristics of the sample, percentage of the participants who met the thresholds for DD and AUD, and the percentage who sought treatment from mental health professionals and other service providers.

The BACE scale was analysed in two steps. First, frequency data for each BACE item was calculated separately for DD and AUD. For each BACE item we calculated the percentage of respondents who reported that they had experienced that barrier to any degree (i.e. the percentage reporting ‘sometimes’ to ‘a lot’) and as a major barrier (i.e. the percentage reporting ‘a lot’). In addition, we assessed if the total BACE score was associated with socio-demographic characteristics of the participants. We report the means and p values for t-test and One-way ANOVA. Further, we conducted post-hoc analysis (Bonferroni) if One-way ANOVA results were significant to indicate which socio-demographic variables were significantly different than other groups. Data analysis was conducted in Stata 13.1 [49], with all of the aforementioned estimates adjusted for complex sampling design.

## Results

Table 1 describes the characteristics of the total sample and the adults who screened positive for DD and AUD. The majority of participants in the sample (60.1%) and the majority of those who screened positive for DD (64.4%) were females, whereas this proportion was only 6.5% among those who screened positive for AUD. The age of the respondents ranged from 18 to 88 years with a mean of 39.8 years (SD 15.4).

**Table 1:** Socio-demographic characteristics of the sample.

Variables	Total sample % (n)*, N = 1983	Screened positive for depression % (n)*, N = 228	Screened positive for alcohol use disorder % (n)*, N = 96
**Sex**			
Male	39.9 (703)	35.6 (71)	93.5 (89)
Female	60.1 (1280)	64.4 (157)	6.5 (7)
**Age (years)**			
18–24	18.4 (296)	18.9 (34)	16.5 (15)
25–59	68.1(1418)	63.6 (156)	71.4 (67)
60 and above	13.5 (269)	17.5 (38)	12.1(14)
Mean (SD)	39.8 (SD, 15.4)	41.0 (SD,16.9)	43.4 (SD,13.5)
**Education**			
Not schooling	13.2 (275)	20.9 (43)	17.2 (18)
Literate/less than primary	14.9 (315)	18.5 (45)	13.6 (12)
Primary	17.6 (360)	17.4 (41)	24.4 (24)
Secondary	41.6 (822)	33.2 (81)	39.1(37)
College /University	12.7 (211)	10.0 (18)	5.7 (5)
**Marital status**			
Single	13.6 (215)	14.3 (27)	12.8 (11)
Married	81.5 (1645)	77.7 (179)	83.5 (80)
Others (widow/divorced/separated)	4.9(123)	8.0 (22)	3.7 (5)
**Caste/Ethnicity**			
Brahmin/Chhetri	47.9 (941)	35.1 (82)	23.7 (25)
Janajati	27.2 (534)	25.8 (65)	31.0 (27)
Dalit	24.9 (508)	39.1 (81)	45.3 (44)
**Religion**			
Hindus	80.3 (1604)	81.8 (179)	75.3 (74)
Non-Hindus	19.7 (379)	18.2 (49)	24.7 (22)
**Occupation**			
Agriculture	62.9 (1299)	67.4 (157)	54.8 (54)
Service/Business	15.9 (293)	9.0 (19)	14.4 (15)
Students/Unemployed	17.4 (315)	18.1 (39)	18.9 (15)
Others	3.8 (76)	5.5 (13)	11.9 (12)

* %, sample weighted percent; n, non-weighted sample size

The adjusted percentage of those who screened positive for DD and AUD in the sample was 11.2% (N = 228) and 5.0 (N = 96) respectively. Details of those who screened positive in the sample have been presented elsewhere [37]. Table 2 presents percentages of participants who had sought and received treatment from a specialist, generalist, or other health care provider for symptoms related to DD and AUD in the last one-year period. For example, 8.1% of those with DD and 5.1% of those with AUD reported that they had received treatment from any provider in the past 12 months.

**Table 2:** Treatment seeking by adults with depression or alcohol use disorder in the past 12 months.

	Depression (N = 228) % (n)*	AUD (N = 96) % (n)*
Receiving treatment in the past year from any providers	8.1 (18)	5.1 (5)
*Type of service providers*		
Generalists (e.g. Doctors and PHC workers)	1.8 (5)	1.3 (2)
Mental health specialists (e.g. psychiatrists, psychologists)	3.6 (9)	0
Others (Traditional healers, religious leaders)	4.2 (8)	4.5 (4)

* %, sample weighted percent; n, non-weighted sample size

Table 3 presents perceived treatment barriers (barrier to any degree or major barrier) and mean scores for each BACE item among adults who screened positive for DD or AUD. For DD, the individual item proportion for experiencing “any degree of barriers” ranges from 55% (item—“concerns about the treatment available e.g. medicine side effect”) to 92.8% (item–“not being able to afford the financial costs involved”). For AUD, the individual item proportion for experiencing “any degree of barriers” ranges from 45.2% (item—“concerns about the treatment available e.g. medicine side effect”) to 96.5% (item–“not being able to afford the financial costs involved”). The proportion of the participants reporting these as major barriers is relatively low i.e. 0 to 24.8% only. The five most frequently reported major barriers for DD were: not being able to afford the financial cost (22.5%); concern that I might be seen as ‘crazy’ (11.0%); dislike of talking about own feelings, emotions or thoughts (10.7%); concern that I might be seen as weak for having mental health problems (9.9%); and having no one who could help me get mental health care (8.3%). Similarly, the five most commonly reported major barriers for AUD were: not being able to afford the financial cost (24.8%); being unsure where to go to get mental health care (13.1%); concern that I might be seen as ‘crazy’ (12.2%); concern that it might harm my chances when applying for jobs (10.9%) and concern that I might be seen as weak for having mental health problems (10.3%).

**Table 3:** Barriers to mental health care among people with DD or AUD who have not received treatment from any providers in the past one year.

	Screened positive for depression (N = 210)			Screened positive for alcohol use disorder (N = 91)		
Barriers to mental health care	Item mean	Barrier to any degree % (n)*	Major barrier % (n)*	Item mean	Barrier to any degree % (n)*	Major barrier % (n)*
Stigma-related barriers						
Concern that I might be seen as weak for having a mental health problem	1.45	90.0 (186)	9.9 (24)	1.40	88.3 (78)	10.3 (13)
Concern that it might harm my chances when applying for jobs	1.20	78.0 (162)	5.2 (16)	1.14	71.6 (63)	10.9 (11)
Concern about what my family might think, say, do or feel	1.15	78.2 (168)	6.5 (16)	0.94	70.0 (61)	3.2 (4)
Feeling embarrassed or ashamed	1.30	87.1 (179)	1.4 (13)	1.34	90.5 (81)	6.1 (7)
Concern that I might be seen as ‘crazy’	1.48	85.3 (183)	11.0 (25)	1.30	79.5 (71)	12.2 (12)
Concern that I might be seen as a bad parent	1.10	82.0 (167)	3.6 (8)	0.77	62.0 (58)	2.0 (3)
Concern that people I know might find out	1.30	84.0 (174)	6.1 (13)	0.98	66.0 (62)	5.4 (6)
Concern that people might not take me seriously if they found out I was having mental health care	1.14	82.2 (170)	4.0 (10)	1.01	74.0 (66)	4.5 (6)
Not wanting a mental health problem to be on my medical records	0.84	58.0 (116)	4.9 (11)	0.60	42.5 (42)	6.1 (6)
Concern that my children may be taken into care or that I may lose access or custody without my agreement	0.89	61.8 (130)	3.4 (8)	0.73	58.0 (51)	0 (0)
Concern about what my friends might think, say or do	1.24	82.5 (174)	6.3 (16)	1.04	73.0 (64)	6.0 (8)
Concern about what people at work might think, say or do	1.19	80.0 (167)	4.5 (9)	1.09	75.1 (67)	6.8 (8)
Non-stigma-related barriers						
Being unsure where to go to get mental health care	1.28	80.3 (165)	8.2 (21)	1.42	83.5 (73)	13.1 (15)
Wanting to solve the problem on my own	1.21	81.5 (168)	7.5 (14)	1.12	74.3 (67)	7.7 (7)
Fear of being put in hospital against my will	0.98	69.1 (144)	3.2 (7)	0.85	69.2 (61)	2.6 (3)
Problems with transport or travelling to appointments	0.90	61.8 (133)	3.8 (14)	0.74	55.4 (47)	4.7 (5)
Thinking the problem would get better by itself	0.93	63.4 (135)	6.1 (13)	0.81	62.5 (57)	1.9 (2)
Preferring to get alternative forms of care (e.g. traditional/religious healing or alternative/complementary therapies)	1.05	74.7 (153)	3.5 (8)	1.15	80.4 (74)	10.1 (11)
Not being able to afford the financial costs involved	1.84	92.8 (197)	22.5 (53)	1.99	96.5 (87)	24.8 (27)
Thinking that mental health care probably would not help	0.84	66.3 (138)	1.9 (6)	0.73	64.9 (58)	0 (0)
Mental health care from my own ethnic or cultural group not being available	0.75	57.0 (113)	0.8 (3)	0.65	51.0 (46)	0.5 (1)
Being too unwell to ask for help	1.30	87.0 (181)	8.1 (17)	1.18	80.1 (70)	7.8 (9)
Dislike of talking about my feelings, emotions or thoughts	1.35	85.4 (179)	10.7 (22)	1.15	75.8 (71)	5.9 (6)
Concerns about the treatments available (e.g. medication side effects)	0.67	55.0 (110)	1.5 (2)	0.52	45.2 (44)	1.4 (1)
Having had previous bad experiences with mental health staff	0.97	67.5 (141)	5.0 (14)	0.80	62.5 (57)	3.0 (3)
Preferring to get help from family or friends	1.12	79.0 (163)	6.2 (12)	0.88	65.2 (58)	0 (0)
Thinking I did not have a problem	1.17	80.0 (166)	7.4 (13)	1.05	74.7 (68)	6.4 (7)
Difficulty taking time off work	0.90	65.2 (132)	2.7 (9)	0.82	63.3 (56)	2.1 (3)
Having problems with childcare while I receive mental health care	0.88	63.0 (129)	2.7 (7)	0.72	57.5 (50)	1.1 (2)
Having no one who could help me get mental health care	1.44	89.3 (186)	8.3 (25)	1.26	80.0 (73)	6.5 (7)

* %, sample weighted percent; n, non-weighted sample size

The mean scores of each BACE item range from 0.67 to 1.84 for DD and 0.52 to 1.99 for AUD. The lowest and highest mean scores for both DD and AUD are for the same barriers, that is, concerns about the treatments available and not being able to afford the financial costs involved, respectively. There was no significant association between BACE total score and treatment seeking (P = 0.361) among participants with DD and AUD.

Table 4 presents results from the t-test and one-way ANOVA. Except occupation, there were no significant differences in mean score for barriers to treatment (BACE) among those with DD or AUD by socio-demographic characteristics. Occupation was significantly associated with mean score of barriers to treatment (BACE) for DD (P = 0.015). Post-hoc comparison using the Bonferroni indicated that the mean score of laborers and people with low earning (mean, 46.5) was significantly higher than that of participants who selected ‘service/business’ (mean, 32.0, P, 0.029) and ‘unemployed/students’ (mean 30.1, P, 0.011) for their occupation. However, the mean score of those who selected ‘agriculture’ (mean, 33.5, P, 0.070) did not significantly differ from the mean score of laborers or people with low earning.

**Table 4:** Comparisons of mean score on barriers to care evaluation with socio-demographic characteristics among adults with depression or AUD who have not received treatment from any providers in the past one year.

Socio-demographic characterizes	Depression		Alcohol Use Disorder (AUD)	
	Mean (95% CI)	*p*	Mean (95% CI)	*p*
**Sex**				
Male	32.5 (29.0–35.9)	0.491	30.2 (27.8–32.6)	0.785
Female	33.9 (31.9–36.0)		28.5 (16.5–40.5)	
**Religion**				
Non-Hindu	35.8 (32.2–39.3)	0.164	32.5 (27.8–37.2)	0.244
Hindu	32.9 (30.9–34.8)		29.3 (26.6–32.0)	
**Age**				
Up to 24	30.1 (25.4–34.9)	0.203	33.0 (26.5–39.6)	0.333
25–59	34.6 (32.5–36.6)		29.0 (26.4–31.6)	
60+	32.7 (29.1–36.3)		32.8 (26.2–39.4)	
**Education**				
No schooling	31.5 (27.8–35.2)	0.697	27.1 (20.9–33.3)	0.681
Primary or less than primary	34.3 (31.8–36.8)		30.6 (26.6–34.7)	
Secondary	33.7 (30.0–37.5)		31.2 (27.6–34.8)	
College /University	33.1 (28.7–37.5)		28.0 (19.6–36.4)	
**Marital status**				
Single	30.4 (24.9–36.0)	0.085	32.4 (25.6–39.2)	0.230
Married	34.4 (32.6–36.2)		30.0 (27.4–32.6)	
Other (widow/divorced/separated)	29.0 (23.7–34.4)		25.5 (20.1–30.9)	
**Caste/Ethnicity**				
Brahmin/Chhetri	33.5 (31.2–35.9)	0.878	29.6 (26.0–33.2)	0.414
Janajati	34.1 (30.4–37.7)		32.5 (28.2–36.9)	
Other	32.8 (29.9–36.0)		28.5 (25.0–32.5)	
**Occupation**				
Agriculture	33.5 (31.4–35.6)	**0.015**	28.8 (25.5–32.0)	0.447
Service/Business	32.0 (28.0–35.9)		29.2 (22.6–35.7)	
Unemployed/Students	30.1 (25.0–35.2)		31.7 (27.5–36.0)	
Other (laborers etc.)	46.5 (37.0–54.0)		35.0 (27.4–42.5)	

Note: Figures presented in the table are adjusted for the sampling design

## Discussion

To the best of our knowledge, this is the first study conducted among the general population to assess barriers to help seeking among people who screened positive for depressive disorder and alcohol use disorder in Nepal. The prevalence of DD and AUD in the sample is relatively lower than that found in studies conducted with specific groups [31, 50, 51] or populations affected by conflict [30, 32] in Nepal. A possible reason for the lower prevalence rates for DD and AUD found in this study could be the fact that all of the previous studies were conducted with specific populations such as refugees, torture survivors, populations displaced by conflict, or populations affected by conflict. The current study was conducted with a representative sample from the general population.

Overall, the treatment gap reported in the sample for both DD and AUD is very high (91.5% and 94.9%). This is considerably higher than the gap reported in other low-income countries, where 52.6% of persons with DD received any treatment in the past 12 months [5]. Moreover, the treatment gap in Nepal could be even greater in other districts where the availability of mental health services is lower than in Chitwan. The treatment gap reported in this sample was smaller than in China [52], Korea [53] and North India [54], whereas it was larger than that found in a study conducted in 8 developed and 6 less developed countries between 2001 to 2003 [26]. Considering the fact that formal mental health services are limited to a few district or zonal hospitals in Nepal [33], these figures exceed our hypothesized estimates for the baseline measurement. We found that treatment seeking was not only low for biomedical services but also for traditional healing. Traditional healers are commonly considered the first point of contact for treatment of general health problems in most of Nepal [33]; thus we were surprised to note that relatively few people reported that they visited traditional healers for treatment of DD (4.0%) and AUD (4.5%) in the past 12 months.

A large proportion of the participants who had not received treatment in the past 12 months reported that they had experienced all of the 30 barriers to some degree, while within this group only 10–20% reported that they had experienced those as major barriers. The most frequently reported major barriers (>10%) were lacking financial means to afford care; fear of being perceived as crazy; lack of information about treatment places; fear of being perceived as weak for having mental health problems; lack of interest in talking about one’s feelings, emotions or thoughts; and preferring alternative treatment. The major barriers in this study are also consistent with the barriers reported in studies conducted in the United States [55], United Kingdom [56], Nigeria [57] and India [29]. We did not find any association between barriers to care and demographic characteristics except for occupation. Laborers and people with low earning had more perceived barriers for depression care compared to other groups. This finding contrasts with those of previous studies conducted in Nepal where age, gender, caste/ethnicity, or marital status had strong associations with mental health outcome [30, 31, 50, 58, 59]. The lack of association between socio-demographic characteristics and total barriers score (BACE score) can be explained by the fact that mental illness is highly stigmatized in the community, and its services are restricted to few government hospitals located in the big cities or private hospitals. In addition to this, poor mental health literacy (even among educated people) may have added barriers to seeking mental health treatment. There was not much difference in reported perceived barriers between those with DD and those with AUD. For example, lacking financial means to afford care, fear of being perceived as crazy, and fear of being perceived as weak for having mental health problems were the most commonly reported major barriers for treatment of both DD and AUD. On the other hand, lack of information about treatment places and preferring alternative treatment were major barriers to treatment seeking and staying in treatment for AUD, whereas not being interested in talking about one’s feelings, emotions or thoughts was a major barrier for depression. In general, AUD is not considered a health problem in Nepal; this could be the reason many participants reported lack of knowledge about treatment places or preferring alternative sources for treatment of alcohol use disorder. We found that both stigma-related and non-stigma-related barriers were equally reported for both DD and AUD. For example, of the 5 major perceived barriers for DD, 3 barriers were not related to stigma and 2 were stigma-related. Similarly, of the 5 major perceived barriers for AUD, 3 were related to stigma and 2 were not stigma-related.

The findings of this study have several implications for the development and implementation of programs to reduce barriers for mental health care and the treatment gap. First, a comprehensive stigma reduction program should be developed. Prior qualitative studies have highlighted mental health stigma as one of the key underlying factors affecting demand for and access to mental health services not only at the community level, but also at the health facility level in Nepal [59–61]. Therefore, the stigma reduction program should also target stigma at the level of service providers. Research has demonstrated that interventions can improve attitudes and competencies of health care providers [62]. Recently we have initiated a program to reduce stigma among service providers and improve clinical care: Reducing Stigma among Healthcare Providers in Mental Health (RESHAPE-mh, clinicaltrials.gov: NCT02793271). Second, the lack of financial means to afford care was the top most reported barrier for both DD and AUD. In the case of Nepal, this is not a surprise because out-of-pocket payment constitutes more than 60% of total health expenditure including fees levied for consultation, investigation, hospitalization, medicines and other supplies [63], and people living in the remote areas have to pay a significant amount for transportation. Availability and accessibility of services is an important indicator to improve health-seeking behavior of people; therefore, one strategy to minimize out-of-pocket expenses and encourage people to receive treatment could be integrating mental health services within the routine health care system and including basic mental health drugs in the free drugs list. Finally, the results indicate that demographic characteristics do not influence barriers to care. Therefore, population-level approaches and strategies can be used to reduce barriers to treatment and improve access to care.

Our study has some limitations. We found a surprisingly low proportion of participants who screened positive for DD during the early data collection phase. After reviewing possible reasons for the low prevalence of DD reported, we changed the Nepali translation of the term ‘mental health’ to a more locally relevant term, ‘heart mind problem’, in the informed consent and questionnaire instructions after completion of the targeted sample of 1500. There was a significant increase in affirmative responses to the PHQ-9 for the last 500 participants who followed the revised consent and instructional material. It is likely that we have underestimated the prevalence of depression in this study. This will be further explored in a separate publication, currently being prepared. Second, there was a significantly high proportion of female participants (59.8%) in the study whereas the proportion of female population of Chitwan district is 51.9% only [40]. The large proportion of females in the sample could be explained by a high out-migration rate among men in Chitwan [64]. Hence, generalizability of the findings may be limited among those adults who were not in the district during the survey. Finally, the BACE scale we used to measure barriers to care has not been validated in Nepal.

## Conclusion

Despite the availability of mental health services in both public hospital and medical colleges, the treatment gap for DD and AUD is high in Chitwan, especially in the primary health care setting. This indicates that the treatment gap is likely to be even more pronounced in other districts where formal mental health services are non-existent. This study revealed the perceived barriers for seeking mental health services likely to contribute to the large treatment gap. The major reported barriers for treatment were lacking financial means to afford care, fear of being perceived as weak for having mental health problems, fear of being perceived as crazy, having no one to help in seeking mental health care, and being too unwell to ask for help. However, there was not much difference between stigma- and non-stigma-related barriers, and the perceived barriers also did not differ by socio-demographic characteristics and type of mental health problem. These results warrant immediate efforts to address barriers to mental health treatment. The results in this study may be useful for policy makers and mental health professionals in developing a strategy to minimize barriers to care and the treatment gap. Except occupation, socio-demographic characteristics did not appear to be related to barriers to care, supporting the possibility of pursuing general population-wide approaches to promoting service use. Mental health services should be integrated within the routine health care system to make sure that basic services are available and accessible, and a strategy should be developed to reduce stigma associated with mental health and improve clinical care.

## Supporting information

S1 PRIME Publication and data Policy_May 2017. (DOCX)
