# Supplementary material for: Treatment gap and barriers for mental health care: A cross-sectional community survey in Nepal
Source: PLoS One. 2017 Aug 17;12(8):e0183223. doi: 10.1371/journal.pone.0183223 (PMC5560728; doi:10.1371/journal.pone.0183223)
Supplement: S1 PRIME — (DOCX) [file pone.0183223.s001.docx]

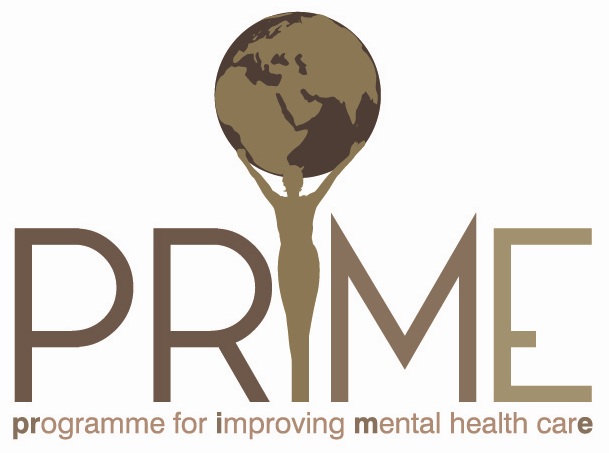


**programme** **for improving** **mental** **health care**

# Data and Publication Policy

Version: May 2017

Related documents:

DFID Research Open and Enhanced Access Policy v1.1 (Jan 2013); DFID Research Open and Enhanced Access Policy v1.1: Implementation Guide; most recent version of the DFID-UCT Contract (PO 5248)

Contents

[Data and Publication Policy 1](#_Toc481587887)

[1. Introduction 4](#_Toc481587888)

[2. Data storage 4](#_Toc481587889)

[2.1 PRIME data storage during the life of PRIME 4](#_Toc481587890)

[2.2 PRIME Data anonymization 5](#_Toc481587891)

[2.3 PRIME data storage after PRIME completion 6](#_Toc481587892)

[2.4 Metadata 6](#_Toc481587893)

[3 Access to data 7](#_Toc481587894)

[3.1 PRIME collaborators 8](#_Toc481587895)

[3.2 Non-PRIME parties 8](#_Toc481587896)

[4 PRIME outputs 11](#_Toc481587897)

[4.1 Journal publications 11](#_Toc481587898)

[4.2 Presentations 14](#_Toc481587899)

[4.3 Policy briefs 15](#_Toc481587900)

[4.4 Books or book chapters 15](#_Toc481587901)

[4.5 PRIME-produced resources 15](#_Toc481587902)

[4.6 Audiovisual material 15](#_Toc481587903)

[4.7 PRIME website 15](#_Toc481587904)

[5 Acknowledgments, disclaimers and additional considerations 15](#_Toc481587905)

[6 Duration and review of policy 16](#_Toc481587906)

[Appendix A: Intention to Publish form – PRIME authors 17](#_Toc481587907)

[Appendix B: Expression of interest/data request form 19](#_Toc481587908)

[Expression of interest/data request form 19](#_Toc481587909)

[Appendix C: Data use agreement 21](#_Toc481587910)

[Appendix D: Biomedical Journals:Ethical Considerations in the Conduct and Reporting of Research: Authorship and Contributorship 29](#_Toc481587911)

[Appendix E: Checklist for publishers/journal access policies and data availability requirements 31](#_Toc481587912)

[Appendix F: DFID Research Open and Enhanced Access Policy 31](#_Toc481587913)

[Appendix G: DFID Research Open and Enhanced Access Implementation Guide 32](#_Toc481587914)

## Introduction

This guideline addresses data storage, data access/sharing, and publication procedures during the life of PRIME and beyond. It is intended to promote open and enhanced access to PRIME data and outputs in accordance with the [DFID policy](https://www.gov.uk/government/uploads/system/uploads/attachment_data/file/181176/DFIDResearch-Open-and-Enhanced-Access-Policy.pdf), and a fair and transparent process for publishing outputs from PRIME to ensure the timely production of high quality research outputs and to build capacity of junior researchers. It is based on the assumption that everyone in the PRIME collaboration will gain by a fair system of sharing these research assets within the collaboration during the life of PRIME. Furthermore, given the wealth of PRIME data and the potential impact of these data, future access privileges given to parties outside the PRIME collaboration will aid in realising the full impact of the collaboration’s efforts. These opportunities for non-PRIME parties will mainly be available once PRIME has been completed, although access to certain datasets may be possible before this time. See section 3.2. Thus, this policy aims to maximise PRIME outputs and impact, through collaboration amongst PRIME partners, particularly encouraging early career individuals, and through collaboration between PRIME partners and external parties.

Under each heading, guidelines will be detailed for two time periods, namely: (1) during the life of PRIME and (2) 0-7 years after the PRIME project has been concluded. (This policy should be revised towards the end of the 7 years to detail the approach to these matters beyond this period.) Within these two time periods, two issues wil be addressed: (1) data storage and access to data and (2) the procedure to be followed in producing actual PRIME outputs, such as peer-reviewed publications, conference reports, book chapters, policy briefs, media reports, etc. Data analysis can include primary and secondary analysis for such outputs. Guidelines for student involvement are also provided in section 4.

This guideline does not apply to indirect PRIME outputs, i.e. outputs not utilising PRIME data. These are any outputs by collaborators which do not use PRIME data but may be related to the overall aims of PRIME. Examples include editorials written by one or more PRIME partners on a subject related to PRIME’s objectives of scaling up mental health care in low resource settings, or outputs published by PRIME partners that report on data from another project which has similar objectives, and clear synergies with PRIME. These indirect outputs will be archived by PRIME during the life of the collaboration and included in reports to DFID under this separate heading.

## Data storage

### PRIME data storage during the life of PRIME

PRIME data are owned jointly by all PRIME partners, in accordance with the legal agreement reached between the partners; thus all partners may access PRIME data. In certain instances, this access will be facilitated by the data management team. Data will be collected by the country teams and maintained in secure storage, e.g. locked cabinets, password protected PCs. It is the responsibility of the country teams to ensure that electronic databases are regularly backed up with a back-up stored in a separate geographical location. Once data is captured electronically a copy should be sent to UCT for central storage on the UCT intranet. Where data is collected by handheld devices such as the Mobenzi system, it will be available on a common server accessible by the Data Manager and other members of the data management team. Country-specific data will be available to that country’s Project Manager and Project Co-ordinator, with access to other countries’ data being provided on request. The Mobenzi data will also be downloaded onto the UCT intranet daily during data collection, and backed up on the data management team members’ password-protected computers. A local copy should be kept by each country and a back up copy will be kept on the UCT intranet once the data is cleaned. Data should be stored for 7 years after the completion of the project as per our contractual arrangements with DFID.

### PRIME Data anonymization

It is a core responsibility of PRIME to ensure appropriate confidentiality while collecting the information provided by the participants in its various research studies. All participants of PRIME studies have been assigned participant ID, which uses a plain no. in combination with the code of the place or mental health condition of the cohort group. PRIME has collected some direct and indirect identifiers such as interviewees’ full name, age, sex, education level, residence, designation (of service providers), telephone/mobile numbers, details of significant others (of people receiving PRIME interventions) however it has been ensured that these direct identifiers has not been recorded with the study data. All physical copies of identifiable information are kept separate from the data in locked cabinet and the soft copies in password protected folders in a secure computer, including audio-recordings of all interviews. These identifiable information is accessible only to core research team members of PRIME who are directly involved in data management. Where follow up is required, field data collectors are provided a list of participants with their names, ID numbers and contact details by the data management team.

Apart from above mentioned we will ensure following additional steps to ensure confidentiality and anonymity of the data shared with other researchers

*Quantitative data*

All quantitative data collected with handheld devices will use participant IDs rather than names to identify participants. On receipt of data, UCT data manager will check the datasets for anonymity prior to data cleaning, archiving and data sharing. Additionally, the UCT will remove village identifiers and add noise to any GPS data so that it is impossible to identify the individuals from their GPS data. In addition, GPS data will only be available for data sharing when there is a clear rationale as to why the authors need to analyse this data.

*Qualitative data*

All interview, transcription and translation files will saved with the Patient Identification number of the participant, diagnosis and treatment category and not using patient’s name by the country teams. During transcription and translation of interviews, patient’s and care giver’s name and address will be removed from all interviews and replaced with a placeholder such as [Participant 1] or [XXXX]. Qualitative data management team will check each transcript to ensure that any direct or indirect identifiers are not included in the same. Only the anonymised data will be sent to UCT for archiving and data sharing. The UCT data manager will check the interview transcripts for anonymity prior to archiving and subsequent data sharing.

### PRIME data storage after PRIME completion

Once PRIME has been completed, the data will remain hosted by UCT on the university intranet. After data locking, the data will be prepared for archiving by the data management team at UCT and LSHTM, namely Emily Baron, Deepak Soowamber and Sujit Rathod, after which the final versions will be uploaded onto the intranet. This will be managed by the data management team at UCT, currently Emily Baron and Deepak Soowamber and overseen by the PRIME CEO and Research Directors, together with the data management oversight from Dr Sujit Rathod at the LSHTM. For data sharing procedures for non-PRIME parties, see section 3.2. These data will include all audiovisual (final versions), quantitative and qualitative data collected during PRIME. The data will be stored for 7 years after the completion of PRIME and a decision will be taken regarding ongoing data storage towards the end of that period.

### Metadata

The metadata, namely details of protocols, summaries of datasets, questionnaires, as well as direct, indirect and planned PRIME outputs will be stored or recorded as described below. (See Table 1 below.) The datasets will be stored by study design, and by country. All questionnaires related to the datasets will be made available, as well as the corresponding codebooks.

A searchable list of direct completed and planned outputs, by category, will be made available on the PRIME website. The data management team will update the published output list monthly, and the planned output list as data request or intention to publish forms are approved.

The metadata will also be deposited on the R4D website, utilising their online submission process. Peer-reviewed publications’ metadata with hyperlinks should be submitted as soon as possible, and the pdf files of these outputs within 6 months of publication (see section 4.1.4). The metadata of non-peer reviewed PRIME outputs should be updated on the R4D website at least once every 6 months.

**Table 1 Metadata: during PRIME and after**

|  | Metadata/output logs | During PRIME | After PRIME (0-7 years) |
| --- | --- | --- | --- |
| 1. | Datasets:   - Study protocols - Questionnaires - Codebooks - Access details | - As cleaned, final datasets become available (as far as possible by 12 months after data locking for that complete dataset, ie endline data for that study included) - On PRIME and R4D websites | - Available for at least 7 years after PRIME work concluded - On archived PRIME website and R4D website |
| 2. | Direct PRIME outputs | - Collected as part of usual reporting * - Reported in annual PRIME reports - Listed with hyperlinks on PRIME and R4D websites - Posted on the PRIME website if possible | - Logged with UCT data management team as part of data access agreement - Listed with hyperlinks on PRIME and R4D websites |
| 3. | Indirect PRIME outputs | - Collected as part of usual reporting* |  |
| 4. | Planned PRIME outputs | - Title and abstract on PRIME website? - ‘Expiry date’ by which output must be completed or title may be taken over by other authors | - Title and abstract on PRIME website - ‘Expiry date’ by which output must be completed or title may be taken over by other authors |

* The PRIME Management Team at UCT will maintain a central database of all PRIME outputs collected as part of the PRIME quarterly reporting during the life of the collaboration. These include planned outputs as well as those which are being drafted or have been published.

## 3 Access to data

This section will deal with data access and sharing procedures. For these issues, there are 3 time periods to be kept in mind, the first 2 being those mentioned in section 1, namely during the life of PRIME and 0-7 years after the project completion. A further time period comes into play regarding data access for non-PRIME partners. According to the [DFID Research Open and Enhanced Access Policy v1.1](https://www.gov.uk/government/uploads/system/uploads/attachment_data/file/181176/DFIDResearch-Open-and-Enhanced-Access-Policy.pdf), data should be made available on request from 18 months after data collection completion. Since this policy came into effect in 2013 for projects that began after November 2012, PRIME is not bound by this policy. PRIME will endeavour to make complete, final datasets (ie, with endline data included) available within 18 months of data locking and this will apply to each study design separately. This will occur within 18 months of final data collection for a study design. For example, 18 months after final data cleaning and locking for the cohort study (in all countries) data will available on request, subject to approval by the PRIME team.

As datasets become available, their availability and access procedures will be advertised on the PRIME website, the Mental Health Innovations Network website, across our networks, student groups (see section 4) and on social media (Twitter and facebook). PRIME collaborators will follow the procedure described in the 2014 version of this policy, using the intention to publish form.

### 3.1 PRIME collaborators

During the life of PRIME, the data management team will have access to all datasets. All country teams will have access to their own data, as well as those collaborating on country-specific papers. Access to the relevant aspects of cross-country data will be given to those collaborating on these papers, according to the PRIME approved publication list.

Once PRIME has been completed, PRIME country teams will retain their cleaned datasets, with duplicate datasets being stored at UCT as described. Further access to data will be facilitated by the data management team in accordance with the publication procedure outlined in section 4.1.2.

### 3.2 Non-PRIME parties

The procedure described below will apply after PRIME completion, and, where possible, for PRIME research where data locking has been completed for 18 months or longer, according to the [DFID policy](https://www.gov.uk/government/uploads/system/uploads/attachment_data/file/181176/DFIDResearch-Open-and-Enhanced-Access-Policy.pdf). Parties interested in accessing PRIME data for publications, conference proceedings, reports, policy briefs, etc will be directed to the website to compare their planned output with the published and planned outputs on the PRIME website. Once they have ascertained that their planned output has not already been planned or published, they will fill in a data request form (see appendix B) on the PRIME website. On receipt of the form, the UCT data management team will compare the request and planned output with the completed PRIME outputs. Should the team find that the output has been published, they will inform the applicant that their request is denied, citing the appropriate publication. However, if the applicants aim to replicate methodology and verify findings, then this request should be considered by the PRIME team.

If the proposed output has not been published or the applicants plan to replicate a methodology or verify findings, the team will compare the request to the planned outputs list. If the planned topic is similar to an already-claimed topic, the team will email the original authors (PRIME/non-PRIME) of the topic, asking them to indicate whether they are still interested in the topic and requesting a timeline. Topics may only be reserved for 6 months, after which the topic will become available again unless significant progress or proof of submission can be shown. In cases where interested authors apply and the topic has been reserved, they will be asked to check the list again once the 6 month period has elapsed at which time they may send a further enquiry. Should the topic requested by the authors be available, the data request form will be sent to the PRIME team for comment (2 week window for comments).

Comments from the team will then be sent to the applicants who will be given 2 weeks to respond. They can submit an amended abstract and request form, or a reply motivating their original plan should there be any requests from the PRIME team for changes. Once these have been resolved and access has been approved, the applicants will be asked to sign an appropriate data use agreement (see appendix C). This data use appropriate will include: (1) agreed timelines; (2) open and enhanced access recommendations (to be adhered to as far as possible); (3) confidentiality and agreement to not further disseminate the data and (4) the inclusion of at least one PRIME author, to be decided by the PRIME team depending on which data is requested according to (a) country: cross-country data (key cross-country partners and others as appropriate) or country-specific data (country teams) and (b) specific study design (partners who played an integral role in a specific aspect of PRIME). Once the appropriate data use agreement has been signed by all authors of the planned output, the UCT data management team will send the authors the required data. Authors should allow at least 2 weeks for the data team to prepare the data.

Should disagreements arise among the authors and the PRIME team, once the appropriate data use agreement has been signed, every effort will be made to resolve the issue. If this fails, the matter will be referred to the PRIME leadership (Profs Lund, Tomlinson and Patel), who will attempt to resolve the matter. Should this not be possible, the matter will come under arbitration according to the UNCITRAL Arbitration Rules (<http://www.uncitral.org/uncitral/en/uncitral_texts/arbitration/2010Arbitration_rules.html>). The following steps will be adhered to: (1) one of the parties will begin the process by sending the other party/parties a notice of arbitration; (2) the party/parties receiving the notice will respond; (3) the parties involved may appoint a representative to assist them; (4) an appointing authority will be chosen at this stage, unless this has already been agreed in the appropriate data use agreement; (5) ‘impartial and independent’ arbitrator(s) will be appointed – either one arbitrator or 3 arbitrators according to the parties’ preferences at the time or prior agreement; (6) the appointment(s) may be challenged by any of the parties involved; (7) the proceedings will include a statement of claim by the party/parties initiating the process, and a statement of response by the other party/parties involved and (8) the matter may be settled or terminated before the final decision if the parties have come to an agreement, or the outcome will be decided by the arbitrator(s). For further details on the arbitration process, see the UNCITRAL website hyperlink above.

**Figure 1 Process for data access – non-PRIME parties**

## 4 PRIME outputs

Procedures related to a number of different outputs are presented below. The DFID policy encourages the production of outputs which are easy to access even in areas with low bandwidth internet access and limited connectivity. All digital outputs, including the website, images and pdf files should be designed to facilitate download of the outputs in such areas. See page 2 of the implementation guide in appendix G for further information.

In the spirit of capacity building, student authorship will be encouraged. This may be facilitated through either a PRIME collaborator or a non-PRIME party. The data access procedures described above should be followed. Extra effort will be made to advertise the PRIME datasets to students. Certain programmes will be targeted, namely: the PhD programme offered by the Department of Psychiatry at Addis Ababa University, the MSc in Global Mental Health at the London School of Hygiene & Tropical Medicine, PhD and postdoctoral fellows in the African Mental Health Research Initiative (AMARI) programme and the MPhil in Public Mental Health offered by the Alan J Flisher Centre for Public Mental Health at the University of Cape Town.

### 4.1 Journal publications

#### 4.1.1 Authorship – PRIME

The authors of a paper and the sequence of authorship should reflect the relative contribution of each individual to the writing of the paper as well as the design, analysis and conduct of the study. In keeping with the Uniform Requirements for Manuscripts Submitted to Biomedical Journals: Ethical Considerations in the Conduct and Reporting of Research: Authorship and Contributorship (Appendix D), authorship credit should be based on:

1. Substantial contributions to conception and design, **or** acquisition of data, **or** analysis and interpretation of data; and
2. Drafting the article or revising it critically for important intellectual content; and
3. Final approval of the version to be published.

Authors should meet all three of the above conditions. All persons designated as authors should qualify for authorship, and all those who qualify should be listed.

The first author should be the individual who is responsible for the first draft of the paper, in addition to taking overall responsibility for the other aspects of the process including research design or data analysis. For cross-country papers, at least 2 country team authors should be invited to contribute to the paper. For country-specific papers, at least 1 cross-country author should be invited to contribute.

External authors may contribute to PRIME publications, including taking the part of the lead author, providing they are identified in the *Intention to Publish* form and this guideline is followed. We encourage long term collaborators to complete the *Expression of Interest* form on the PRIME website which can be circulated to the consortium to inform them of new collaborators.

Principles of capacity building should be inherent in the choice of authors. Junior members of PRIME should be encouraged to take the lead on outputs and thus qualify for first authorship. However, it is important to ensure that the authorship group has the necessary technical skills to complete the paper. Where these are not available within the PRIME group, external authors should be brought in, and identified in the *Intention to Publish* form outlined in the process below.

#### 4.1.2 Procedure for writing and submitting a publication – PRIME

A potential publication list will be drafted by the PMG with inputs from the consortium with possible outputs of PRIME, both inter- and intra-country. This should be seen as a fluid document which will provide a guideline for collaborators but should not limit the scope of possible publications from PRIME. The potential publication list will be circulated quarterly and available on request from the Erica Breuer.

Country partners will have preferential access to their own country data, and should be encouraged to take the lead on their country-specific papers. This does not exclude cross-country partners working with data from a single country or country partners working with cross-country data. Cross-country partners wishing to write an article based on country data should discuss this with the relevant PRIME country teams where the data was collected and any other collaborators they wish to include.

The process for writing and submitting a publication is set out in Figure 1. Once an outline of the paper and a list of authors has been established, the lead author should submit an *Intention to Publish* form (Appendix A) to the PRIME Administrator, Gillian Hanslo (Gillian.hanslo@uct.ac.za). Once it has been received, the PMT will check the intended paper against the Intention to Publish Database to determine if there is any potential duplication with an existing intended publication, and how it aligns with other papers on the potential publication list. This information will be relayed to the author. The intention of this step is to coordinate the various PRIME outputs and in most instances it is expected that there will be minimal or no changes to the Intention to Publish form that has been submitted.

Following this, the Intention to Publish form will be sent out to the PMG who are invited to comment on the Intention to Publish, within **two weeks** of the receipt. If the author does not agree with the PMG feedback, the author will be asked to submit a motivation to the PMG who may decide on how to take the matter forward.

Any dispute around authorship or the process will be handled by the Prime Management Group, in accordance with general PRIME policies for managing disputes between partners as specified in the subcontracts. In situations where a publication is not submitted according to the timetable or if more than one author wants to take the lead on a specific publication and they are not able to come to an agreement, the PMG have the authority to adjudicate and their decision may include the appointment of a new or lead author**.** It should be noted that the timetable for writing papers will be generous, open to negotiation and will include an important component of capacity development.


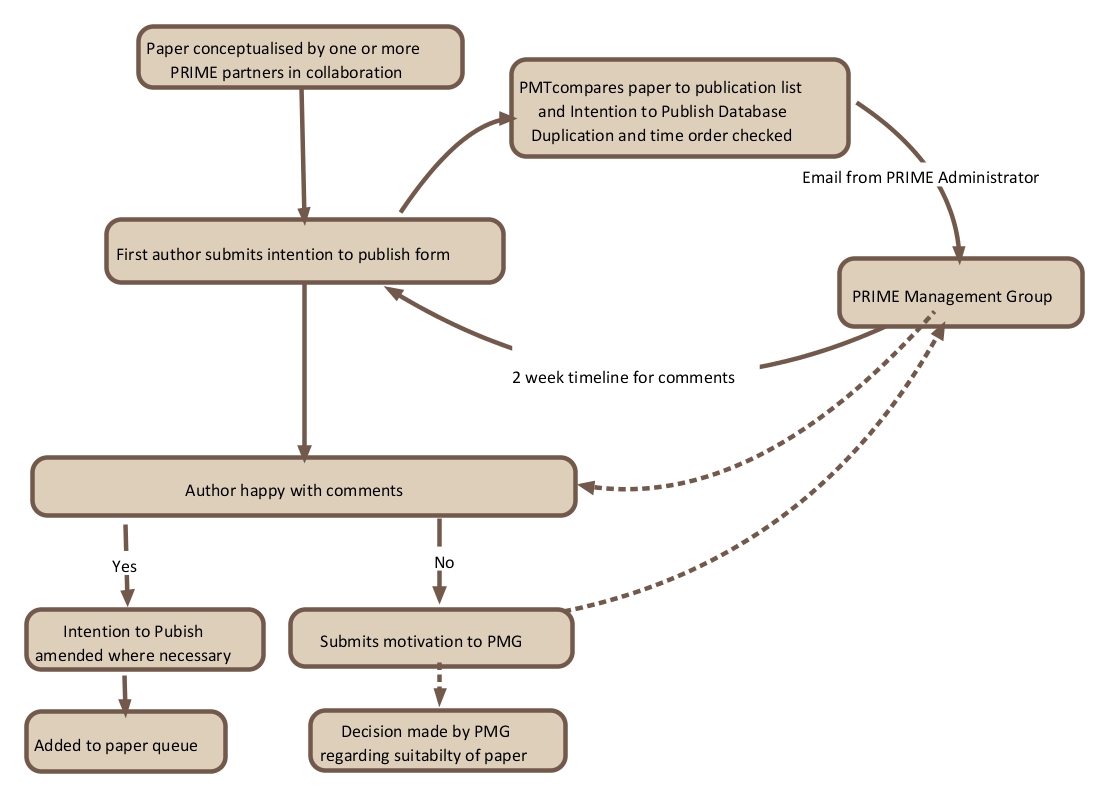


Figure 1. PROCESS FOR SUBMITTING THE INTENTION TO PUBLISH FORM

Designated co-authors who do not provide the requisite intellectual input, according to the above authorship criteria within a one month period of requesting that input at each stage of the process (e.g. paper outline, data analysis, first draft, final draft) will be asked to withdraw as co-authors. This shall initially be done informally by the first author but may require adjudication by the PMG as above. The updated PRIME paper queue will be available on the PRIME website for all partners to access. Once the Intention to publish form has been submitted, the process towards publication will be monitored using the outputs section of the quarterly reports.

#### 4.1.3 Procedure for writing and submitting a publication – non-PRIME

Non-PRIME authors will access data as described above in section 3.2. Once accepted by the PRIME management team, their proposed title and abstract will be added to the planned output list posted on the PRIME website. The authors will be asked to submit the completed output or hyperlink for the output to the UCT data management team. (See section 2.3 above.) As with all other PRIME outputs, both PRIME and DFID should be acknowledged in the standard manner on all publications produced by non-PRIME partners (see section 5).

#### 4.1.4 Choice of journal and publishing strategies – PRIME and non-PRIME

PRIME is committed to open access publication as *per* DFID’s policy and implementation guide (see appendices F and G). Although PRIME collaborators are not contractually obliged to adhere to this policy, as it only applies to research grants awarded from November 2012, we will aim to adhere to this policy as far as possible. Wherever feasible, authors should publish in open access format, whereby an article is freely available on a journal website (gold open access). To enable this it may be necessary to apply for an open access fee waiver or for reduced fees from the journal which supports open access publication. Alternatively, where possible, authors should consider applying for additional funding from their institutions to support this. All recipients of PRIME data will be encouraged to publish in open access format. Furthermore, DFID encourages PRIME authors to choose journals which allow the researchers, or their institutions, to retain copyright of the output.

Another open access option is so-called green open access. This option may be selected if gold open access is not possible. With this option, authors should self-archive the output, i.e. publish it on the PRIME website and the R4D website within 6 months of initial publication. Authors should ensure that they have the necessary permissions to do so. The output versions should be final drafts *after* completion of the peer review process. To check a journal’s policy on self-archiving, search the [SHERPA/RoMEO database](http://www.sherpa.ac.uk/romeo/search.php), or contact the journal editorial team. When considering the green open access option, authors are encouraged to identify journals or publishers which give preferential access low- and middle-income country users. The World Health Organization’s HINARI Access to Research in Health Programme provides free or low cost access to certain journals and resources to institutions in selected low- and middle-income countries. See the HINARI website for a list of [participating publishers](http://extranet.who.int/hinari/en/partners.php?category=publisher).

### 4.2 Presentations

#### 4.2.1 PRIME collaborators

Any collaborators wishing to present any form of research findings based on the PRIME findings at an academic conference should submit an *Intention to Publish* form prior to submission of abstracts using the same process as for peer reviewed journal articles described above. Authors need to take responsibility to ensure that conference presentations do not prejudice successful publication according to conference and journal policies. In addition, if any PRIME partner wishes to present PRIME data at a conference before it has been published, they should seek the consent of the other authors involved in publishing that data.

A standard PRIME slide will be provided for conference presentation purposes with the appropriate logos, acknowledgements and disclaimers.

#### 4.2.2 Non-PRIME parties

Non-PRIME parties should follow the data access procedures for conference presentations. Should they wish to publish a peer-reviewed article as well as present the findings at a conference, both should be mentioned in the expression of interest/data access form. As with all other PRIME outputs, both PRIME and DFID should be acknowledged in the standard manner on all conference presentations by non-PRIME partners (see section 5).

### 4.3 Policy briefs

Access to data for policy briefs is as per guidelines for PRIME or non-PRIME parties as appropriate. These should be sent to the PRIME team for them to deposit on the R4D and PRIME websites. Authors are encouraged to make these available in the languages of their area/country, ie all translations that will be utilised in developing the output.

### 4.4 Books or book chapters

Prior to publication, authors should request full or partial open access for the book or chapter. Should this not be possible, DFID recommends that the authors arrange with the publisher to buy back copies to distribute. Whether open access or not, a 500 word synopsis and table of contents should be deposited on the R4D and PRIME websites once the book is published.

### 4.5 PRIME-produced resources

Such resources should be licensed through Creative Commons using the ‘Attribution’ licence CC-BY, as appropriate. For more information, refer to the [DFID Research Open and Enhanced Access Policy v1.1: Implementation Guide](https://www.gov.uk/government/uploads/system/uploads/attachment_data/file/181177/DFIDResearch-Open-and-Enhanced-Access-Implementation-Guide.pdf) and the [Creative Commons website](https://creativecommons.org).

### 4.6 Audiovisual material

All final versions of the audiovisual material produced by the PRIME team should be made freely available online. The PRIME documentary clips and films are available on YouTube, with links from the PRIME website. These links and metadata will also appear on the R4D website.

### 4.7 PRIME website

The PRIME website will be managed by the PRIME team for the duration of the project and for seven years after PRIME. Should there be no resources for the site to continue after this time, the website and all its contents will be archived. Should the PRIME website address change at any time, the team will notify the R4D website staff.

## 5 Acknowledgments, disclaimers and additional considerations

All direct outputs of PRIME work by PRIME or non-PRIME authors should contain the following statement in the Acknowledgements (or appropriate) section, in keeping with our contractual arrangement with DFID:

“*This study is an output of the PRogramme for Improving Mental health carE (PRIME). This work was supported by the UK Department for International Development [201446]. The views expressed do not necessarily reflect the UK Government’s official policies.”*

For papers on which WHO staff members are listed as authors, the following disclaimer should be included:

*"X and Y are staff members of the World Health Organization. The authors alone are responsible for the views expressed in this publication and they do not necessarily represent the decisions, policy or views of the World Health Organizatio****n."***

Additional acknowledgements for individuals who have substantially contributed to the study such as site staff, study co-ordinating staff and user groups will be decided by the authors. It should be noted that journals may require written approval from all named persons who are listed in the Acknowledgements for their names to be mentioned.

Staff members of WHO are required to obtain internal clearance on any information products where staff members of WHO are listed specifically as authors. This may take a few weeks to obtain and should be factored into the timetable for final submission.

## 6 Duration and review of policy

The principles of this guideline will apply to the publishing of all PRIME data until 7 years after the formal completion of the project. The PMG will review the guideline at the annual meetings in 2012, 2014 and 2016. The 2016 draft of the guideline will formalise the publication process to be followed after the formal completion of the project. This guideline should then be reviewed in 2018 and at two-yearly intervals, or sooner should any circumstances change.

______________________________

*We would like to thank Prof Graham Thornicroft and the ASPEN and INDIGO-Depression Projects for the use of their Publication Protocol for ASPEN and INDIGO-Depression (January 2011) as a guideline for developing this protocol.*

# **Appendix A: Intention to Publish form – PRIME authors**

**First author:**

**Title:**

**Other authors: (Anticipated) Contribution**

**2**

**3**

**4**

**5**

**6**

**7**

**8**

**9**

**10**

**Sources of data**

**Site:**

**Ethiopia India Nepal South Africa Uganda Cross country n/a**

**Type of analysis: Primary Secondary**

**Phase: Inception Implementation Scaling up**

**Brief description of paper (including aims and methods):**

**Stage of paper: Planning Draft preparation**

**Proposed completion date**

**Proposed dissemination**

***Conference***

**National International Funding secured**

**Name of conference: Date of conference: Place of conference:**

***Publication***

**Target date for submission: Target journal:**

**Date log form updated: Additional Comments:**

# Appendix B: Expression of interest/data request form

# Expression of interest/data request form

**1. Name and Surname** *

**2. Country of Origin** *

**3. Occupation** *

**4. Email Address** *

**5. How did you find out about PRIME?** *

1. Website
2. Colleague
3. Journal Articles
4. Conference Presentations
5. Media (Newspapers, TV, Radio etc)
6. Other:

**6. Current Affiliation**

**7. I am interested in:**

1. Accessing PRIME data (CONTINUE to question 8)
2. Implementing the PRIME strategy in my area/country (SKIP to 13)
3. Working with the PRIME team (SKIP TO no. 17 – usual EOI form)
4. Using the PRIME images (SKIP TO no. 24)
5. Using the PRIME film (SKIP to no. 25)
6. Other _______________________________________

**8. I would like to use PRIME data for: (more than one option may be selected)**

1. Peer-reviewed journal publication
2. Conference presentation
3. Policy brief
4. Student dissertation
5. Media article
6. Other ____________________________________

**9. Which data would you like to access?** (Allow this section to be completed multiple times)

*Please go to XXXXXX for details on the datasets currently available, formats and codebooks.*

Data Set:__________________________________________

Requested Sections:_____________________________

**10. Please provide a brief abstract or description of your proposed end product.**

*Before you submit your abstract, please access the planned and published outputs list on the PRIME website (INSERT HYPERLINK TO OUTPUTS LIST HERE) to make sure that this topic has not already been covered.*

**11. Where do you plan to present or publish this product?**

1. Peer-reviewed journal (please provide the journal title)
2. Conference (please provide the conference details)
3. Website
4. University for student dissertation
5. Other publication ______________________

**12. Estimated completion date (END for data request)_______________________**

**13. I would like to implement the PRIME strategy in (area/country/region)________________**

**14. I believe that the PRIME strategy is appropriate for my context because ________**

**15. Please describe the aspects of the PRIME strategy that you would like to implement.**

**16. I would need the following expertise from the PRIME team _________ (END for PRIME strategy)**

17. **Which PRIME team are you interested in working with?** *

1. Ethiopia
2. India
3. Nepal
4. South Africa
5. Uganda
6. Not sure yet
7. Other:

**18. Please summarise briefly your prior discussions or collaboration with any PRIME partners.**

**19. How do you see yourself becoming involved in PRIME? Specifically, how can you contribute to the goals, outputs or impact of PRIME?** *

**20. Do you have funding that could cover your collaboration with PRIME?** * If Yes, please specify source of funding under Other

1. Yes
2. No [Skip to 22]
3. Other:

**21. Please indicate what this funding will cover**

1. Salary
2. Travel
3. Research Costs
4. None
5. Other:

[Skip to 23]

**22. If you do not have funding, please specify how you will fund the time and costs associated with your work on PRIME?**(END working with PRIME team)

**23. PRIME Images:**

*See (INSERT HYPERLINK HERE) for list of PRIME Images*

Which images are you interested in using?

1. To what target audience/demographic will the image be disseminated?
   3. How many readers do you expect? (END interest in PRIME images)

**24. PRIME Films:**

*See (INSERT HYPERLINK HERE) for list of PRIME films*

Which film/video are you interested in using?

Where will the video/film be screened/disseminated?

To what target audience/demographic will the video/film be disseminated?

How many viewers do you expect? (END interest in PRIME film)

# Appendix C: Data use agreement

**DATA USE AGREEMENT**

ENTERED INTO BETWEEN

UNIVERSITY OF CAPE TOWN (UCT)

Data Provider

AND

Data Recipient

**Contact information for data recipient**

Name :

Mailing address :

Telephone/Fax :

Email :

**Authorised official for data recipient**

Name :

Mailing address :

Telephone/fax :

Email :

Name of Program: Programme for Improving Mental Healthcare.

Description of research project: [SHORT OUTLINE OF THE RESEARCH PROJECT INCLUDING THE RESEARCH QUESTIONS/AIMS AND OBJECTIVES]

Description of data: [*INSERT NAME OF DATASET AND COUNTRIES HERE*]. Details of the sections and variables are outlined in Annex 1.

Effective Date: *[INSERT DATE HERE]*

In consideration of receipt of the above-referenced date, the undersigned data recipient agrees to the following:

1. Ownership. Except as expressly set forth in this agreement, nothing herein shall be construed as granting data recipient any rights in the data. Data provider shall retain all ownership rights in the data.
2. Limited use. The data will be used only for scientific research related to the Program or Project indicated above.
3. Data access. Access to the data will be restricted to data recipient’s researchers who are required to have access for the purpose of carrying out the Program or Project.
4. Non-commercial use. The data are for non-profit, non-commercial use only. No profit or other commercial gain may be obtained through the use of any of the data.
5. De-identification and/or contact with individuals. Data recipient will not attempt to identify any persons related to the data nor contact the individuals who are the subject of the data.
6. Data security. Data recipient will ensure that all data are stored and analysed in a secure environment and adequately protected from access by unauthorised or third parties.
7. No redistribution. Data recipient will not publish the data (online or in any other format) or distribute the data in whole or part ( with or without charge) to any other individual or organisation, providing that the data may be referenced in a peer-reviewed scholarly publication if the data are properly cited as stated in clause 9 below.
8. Reporting of unauthorised use or disclosure. Data recipient will report in writing to data provider any unauthorised use or disclosure of the data within five (5) working days of becoming aware of the unauthorised use or disclosure.
9. Data oration. Data recipient will acknowledge data provider and funder in any publications resulting from the use of the data using “*This study used data from the PRogramme for Improving Mental health carE (PRIME). This work was supported by the UK Department for International Development. The views expressed do not necessarily reflect the UK Government’s official policies.”*
10. Open access. The data should preferentially be published in an open access journal in line with DFID’s Research Open and Enhanced Access Guidelines.
11. Co-authorship. At least one member of the PRIME research programme consortium should be included as an author in peer reviewed publications. These author(s) will be determined by the PRIME research programme consortium.
12. Copies of publication. Data recipient will inform data provider about publications using the data and if feasible send an electronic copy of the publication to *[INSERT RELEVANT EMAIL ADDRESSE(S) HERE]*
13. Term and termination. This agreement is effective as of the effective date above and will continue for 6 months after this date unless significant progress or proof of submission can be shown. Either party may terminate this agreement immediately upon written notification to the other party and without liability for such termination. Upon the termination of this agreement for any reason, data recipient agrees that it will either return or destroy the data as instructed by data provider.
14. Severability. If any provision of this agreement is found to be wholly or partially invalid or unenforceable, the remainder of this agreement shall be unaffected.
15. Waiver. No term or provision of this agreement shall be deemed waived and no breach excused unless waiver or excuse of breach is in writing and signed by the party against whom such waiver or excuse is claimed.
16. Indemnification. The parties agree to defend, indemnify and hold each other harmless from and against any loss, claim or damage arising from the negligent acts or omissions of their own officers, employees, students or agents in the performance of their duties under this agreement.

- Governing Law. Should any disputes arise between the parties in connection with this Agreement or any items not covered by this Agreement, the parties shall use their best efforts to resolve the dispute through the negotiation between the parties.  Any such dispute not satisfactorily settled by the parties shall be settled in accordance with the rules of International Chamber of Commerce in the place of the respondent, namely, in [*INSERT LOCATION OF DATA RECIPENT*] if the respondent is [INSERT NAME OF DATA RECIPENT], or in Cape Town South Africa if the respondent is University of Cape Town.  Nothing herein shall preclude either party from seeking interim equitable relief from a court of competent jurisdiction.  A request by a party to a court for interim relief shall not affect either party’s obligation hereunder to arbitrate.  The award of such arbitration shall be final and binding on the parties and shall not be subject to appeal to any court, and may be entered in the court of competent jurisdiction for execution forthwith.  This Agreement and any arbitration to follow shall be construed and interpreted in accordance with the laws of South Africa.

**Data Provider**

| SIGNED AT ________________ THIS _______ DAY OF _________________ | | | |
| --- | --- | --- | --- |
| For Data Provider | Name | Designation | Signature |

**Data recipient**

| SIGNED AT ________________ THIS _______ DAY OF _________________ | | | |
| --- | --- | --- | --- |
| For Data Recipient | Name | Designation | Signature |

**Authorised official for data recipient**

| SIGNED AT ________________ THIS _______ DAY OF _________________ | | | |
| --- | --- | --- | --- |
| For Data Recipient | Name | Designation | Signature |

Annex 1: PRIME data

| PRIME Dataset | Country | | Sections/Variables included | |  |
| --- | --- | --- | --- | --- | --- |
|  | **Phase 1: Inception** | |  | |  |
|  | **Formative interviews** | |  | |  |
|  |  | Ethiopia | |  |  |
|  |  | India | |  |  |
|  |  | Nepal | |  |  |
|  |  | SA | |  |  |
|  |  | Uganda | |  |  |
| Phase 2: Implementation | | |  | |  |
|  | **Community survey** | |  | |  |
|  |  | Baseline | |  |  |
|  |  | Ethiopia | |  |  |
|  |  | India | |  |  |
|  |  | Nepal | |  |  |
|  |  | Uganda | |  |  |
|  |  | Endline | |  |  |
|  |  | Ethiopia | |  |  |
|  |  | India | |  |  |
|  |  | Nepal | |  |  |
|  |  | Uganda | |  |  |
|  | **Facility detection survey** | |  | |  |
|  |  | Baseline | |  |  |
|  |  | Ethiopia | |  |  |
|  |  | India | |  |  |
|  |  | Nepal | |  |  |
|  |  | SA | |  |  |
|  |  | Uganda | |  |  |
|  |  | Interim | |  |  |
|  |  | Nepal | |  |  |
|  |  | Uganda | |  |  |
|  |  | Ethiopia | |  |  |
|  |  | Endline | |  |  |
|  |  | Ethiopia | |  |  |
|  |  | India | |  |  |
|  |  | Nepal | |  |  |
|  |  | SA | |  |  |
|  |  | Uganda | |  |  |
|  | **Cohorts** | |  | |  |
|  |  | Depression | |  |  |
|  |  | Ethiopia | |  |  |
|  |  | India | |  |  |
|  |  | Nepal | |  |  |
|  |  | SA | |  |  |
|  |  | Uganda | |  |  |
|  |  | AUD | |  |  |
|  |  | Ethiopia | |  |  |
|  |  | India | |  |  |
|  |  | Nepal | |  |  |
|  |  | Psychosis | |  |  |
|  |  | Ethiopia | |  |  |
|  |  | India | |  |  |
|  |  | Nepal | |  |  |
|  |  | SA | |  |  |
|  |  | Uganda | |  |  |
|  |  | Epilepsy | |  |  |
|  |  | Ethiopia | |  |  |
|  |  | Nepal | |  |  |
|  |  | Uganda | |  |  |
|  |  | Maternal depression | |  |  |
|  |  | Uganda | |  |  |
|  | **Case study** | |  | |  |
|  | **Profiles** | |  | |  |
|  |  | Facility profiles | |  |  |
|  |  | Ethiopia | |  |  |
|  |  | India | |  |  |
|  |  | Nepal | |  |  |
|  |  | SA | |  |  |
|  |  | Uganda | |  |  |
|  |  | Community and district profiles | |  |  |
|  |  | Ethiopia | |  |  |
|  |  | India | |  |  |
|  |  | Nepal | |  |  |
|  |  | SA | |  |  |
|  |  | Uganda | |  |  |
|  | **Implementation logs** | |  | |  |
|  |  | Ethiopia | |  |  |
|  |  | India | |  |  |
|  |  | Nepal | |  |  |
|  |  | SA | |  |  |
|  |  | Uganda | |  |  |
|  | **Training and supervision** | |  | |  |
|  |  | Ethiopia | |  |  |
|  |  | India | |  |  |
|  |  | Nepal | |  |  |
|  |  | SA | |  |  |
|  |  | Uganda | |  |  |
| Qualitative evaluation of implementation | | | | |  |
|  | Service users | |  | |  |
|  |  | Ethiopia | |  |  |
|  |  | India | |  |  |
|  |  | Nepal | |  |  |
|  |  | SA | |  |  |
|  |  | Uganda | |  |  |
|  | Service providers | |  | |  |
|  |  | Ethiopia | |  |  |
|  |  | India | |  |  |
|  |  | Nepal | |  |  |
|  |  | SA | |  |  |
|  |  | Uganda | |  |  |
|  | Phase 3: scale up | | | | |
| Implementation logs | | | | | |
|  |  | Ethiopia | |  |  |
|  |  | India | |  |  |
|  |  | Nepal | |  |  |
|  |  | SA | |  |  |
|  | **Facility Profile** | |  | |  |
|  |  | Ethiopia | |  |  |
|  |  | India | |  |  |
|  |  | Nepal | |  |  |
|  |  | SA | |  |  |
|  | **Quality of care study** | |  | |  |
|  |  | Nepal | |  |  |
|  |  | SA | |  |  |
|  |  | Anyone else? | |  |  |
|  | **Assessment of training quality** | |  | |  |
|  |  | Ethiopia | |  |  |
|  |  | India | |  |  |
|  |  | Nepal | |  |  |
|  |  | SA | |  |  |
|  | **Assessment of supervision quality** | |  | |  |
|  |  | Ethiopia | |  |  |
|  |  | India | |  |  |
|  |  | Nepal | |  |  |
|  |  | SA | |  |  |
|  | **MHIS data** | |  | |  |
|  |  | Ethiopia | |  |  |
|  |  | India | |  |  |
|  |  | Nepal | |  |  |
|  |  | SA | |  |  |

# Appendix D: Biomedical Journals:Ethical Considerations in the Conduct and Reporting of Research: Authorship and Contributorship

International Committee of Medical Journal Editors

Accessed at <http://www.icmje.org/ethical_1author.html> [ 7^th^ August, 2011]

Byline Authors

An “author” is generally considered to be someone who has made substantive intellectual contributions to a published study, and biomedical authorship continues to have important academic, social, and financial implications (1). *An author must take responsibility for at least one component of the work, should be able to identify who is responsible for each other component, and should ideally be confident in their co-authors’ ability and integrity.* In the past, readers were rarely provided with information about contributions to studies from persons listed as authors and in Acknowledgments (2). Some journals now request and publish information about the contributions of each person named as having participated in a submitted study, at least for original research. Editors are strongly encouraged to develop and implement a contributorship policy, as well as a policy on identifying who is responsible for the integrity of the work as a whole.

While contributorship and guarantorship policies obviously remove much of the ambiguity surrounding contributions, they leave unresolved the question of the quantity and quality of contribution that qualify for authorship. The ICJME has recommended the following criteria for authorship; these criteria are still appropriate for journals that distinguish authors from other contributors.

Authorship credit should be based on 1) substantial contributions to conception and design, acquisition of data, or analysis and interpretation of data; 2) drafting the article or revising it critically for important intellectual content; and 3) final approval of the version to be published. Authors should meet conditions 1, 2, and 3.

When a large, multicenter group has conducted the work, the group should identify the individuals who accept direct responsibility for the manuscript (3). These individuals should fully meet the criteria for authorship/contributorship defined above, and editors will ask these individuals to complete journal-specific author and conflict-of-interest disclosure forms. When submitting a manuscript authored by a group, the corresponding author should clearly indicate the preferred citation and identify all individual authors as well as the group name. Journals generally list other members of the group in the Acknowledgments. The NLM indexes the group name and the names of individuals the group has identified as being directly responsible for the manuscript; it also lists the names of collaborators if they are listed in Acknowledgments.

Acquisition of funding, collection of data, or general supervision of the research group alone does not constitute authorship.

All persons designated as authors should qualify for authorship, and all those who qualify should be listed.

Each author should have participated sufficiently in the work to take public responsibility for appropriate portions of the content.

Some journals now also request that one or more authors, referred to as “guarantors,” be identified as the persons who take responsibility for the integrity of the work as a whole, from inception to published article, and publish that information.

Increasingly, authorship of multicenter trials is attributed to a group. All members of the group who are named as authors should fully meet the above criteria for authorship/contributorship.

The group should jointly make decisions about contributors/authors before submitting the manuscript for publication. The corresponding author/guarantor should be prepared to explain the presence and order of these individuals. It is not the role of editors to make authorship/contributorship decisions or to arbitrate conflicts related to authorship.

Contributors Listed in Acknowledgments

All contributors who do not meet the criteria for authorship should be listed in an acknowledgments section. Examples of those who might be acknowledged include a person who provided purely technical help, writing assistance, or a department chairperson who provided only general support. Editors should ask corresponding authors to declare whether they had assistance with study design, data collection, data analysis, or manuscript preparation. If such assistance was available, the authors should disclose the identity of the individuals who provided this assistance and the entity that supported it in the published article. Financial and material support should also be acknowledged.

Groups of persons who have contributed materially to the paper but whose contributions do not justify authorship may be listed under such headings as “clinical investigators” or “participating investigators,” and their function or contribution should be described—for example, “served as scientific advisors,” “critically reviewed the study proposal,” “collected data,” or “provided and cared for study patients.” Because readers may infer their endorsement of the data and conclusions, these persons must give written permission to be acknowledged.

# Appendix E: Checklist for publishers/journal access policies and data availability requirements

When selecting a journal for publication, please take note of the following:

1. Open access policy:
   1. Is the journal an open access journal, or does the journal offer an open access option?
   2. If so, how much does the journal charge in article processing fees?
   3. Does the journal offer better rates to authors from LMICs?
   4. Does your academic institution have an agreement with the publisher regarding fees?
   5. If you are a PRIME author, check with your team if there are open access fees available.
2. Green open access:
   1. Does the journal allow authors to self-archive or post the paper online?
   2. If so, in what version may the authors post it – pre-production or post-print?
   3. If so, when may the author post it – after 6 months or 12 months? (DfID prefers 6 months.)
3. Copyright:
   1. Do authors retain copyright?
   2. Or does the journal ask authors to transfer copyright to the journal or publisher? Is this negotiable? (DfID prefers authors to retain copyright.)
4. Data availability
   1. Does the journal have requirements regarding data availability?

If so,

1. when should the data be made available – on publication, on completion of the study?
2. for how long should the data be available – five years, ten years after publication?
3. where should the data be made available – online in a repository, as supplemental files, on request?
4. In what format should the data be?
5. Does this apply to quantitative and qualitative data?

# Appendix F: DFID Research Open and Enhanced Access Policy

https://www.gov.uk/government/uploads/system/uploads/attachment_data/file/181176/DFIDResearch-Open-and-Enhanced-Access-Policy.pdf

(will be appended to the pdf version)

# Appendix G: DFID Research Open and Enhanced Access Implementation Guide

(will be appended to the pdf version)
